# Immune response to racotumomab in a child with relapsed neuroblastoma

**DOI:** 10.3389/fonc.2012.00195

**Published:** 2012-12-20

**Authors:** C. Sampor, M. D. Guthmann, A. Scursoni, W. Cacciavillano, A. Torbidoni, L. Galluzzo, S. Camarero, J. Lopez, M. T. G. de Dávila, L. Fainboim, G. L. Chantada

**Affiliations:** ^1^Department of Hemato-Oncology, Pediatric Hospital Prof. Dr. Juan P. GarrahanBuenos Aires, Argentina; ^2^Laboratorio EleaBuenos Aires, Argentina; ^3^Department of Pathology, Pediatric Hospital Prof. Dr. Juan P. GarrahanBuenos Aires, Argentina; ^4^Laboratory of Molecular Oncology, National University of QuilmesBuenos Aires, Argentina; ^5^Laboratory of Immunogenetics, Jose de San Martin Clinics Hospital, University of Buenos AiresBuenos Aires, Argentina

**Keywords:** immunotherapy, monoclonal antibodies, neuroblastoma, ganglioside, racotumomab

## Abstract

Immunotherapy targeting ganglioside antigens is a powerful tool for the treatment of high risk neuroblastoma. However, only treatment with anti-GD2 antibodies has been used in clinical practice and other options may be pursued. We report the use of racotumomab, an anti-idiotype vaccine against N-glycolyl neuraminic acid (NeuGc)- containing gangliosides, eliciting an immune response in a child with relapsed neuroblastoma expressing the NeuGcGM3 ganglioside.

## Introduction

This is a 4-year-old female patient who presented with diffuse pain in lower limbs leading to walking problems and a 1-month history of intermittent fever and generalized pallor. Physical examination showed right eye proptosis and an abdominal mass. Laboratory tests showed anemia (hemoglobin 7.5 g %) and elevated LDH. The child was hospitalized at another center for evaluation. A CT scan of the abdomen revealed a large calcified abdominal mass originating from left adrenal gland. She was referred to our Hospital for further treatment. At our hospital, neuroblastoma cells were evident at a bone marrow examination. Malignant cells were positive for 1p deletion and showed MYCN amplification. Metaiodobenzylguanidine (MIBG) scintigraphy revealed multiple tumoral foci in skull, spine, and left upper quadrant of the abdominal mass. Urinary catecholamines determination revealed elevated norepinephrine levels and vanillyl mandelic acid (VMA).

Therefore, with a diagnosis of Stage 4 neuroblastoma belonging to the high risk group, chemotherapy was prescribed, including 5 cycles of a standard induction regimen (Matthay, 1999). Evaluation after induction chemotherapy showed progressive disease in the abdominal tumor and in the bone marrow.

A second line regimen including 3 cycles of topotecan and carboplatin was given. A repeated response evaluation revealed persistent bone marrow infiltration, progressive disease in the orbit with intracranial extension, thoracic and lumbar vertebrae as well as a persistent left heterogeneous retroperitoneal mass 10 × 8 × 8 cm and high catecholamine levels. The patient had significant malaise and widespread pain. The disease was deemed refractory to conventional therapy and she was considered for experimental treatment.

## Background

Despite advances in the treatment of pediatric malignancies, cancer is the second most common cause of death in children over 1-year-old in developed countries. In Argentina, it accounts for the third leading cause of death in children preceded by accidents and congenital malformations (Scursoni et al., 2011). Children with primary multifocal, refractory or relapsed malignant solid tumors still have a very poor prognosis. On the other hand, most therapies are associated with significant toxicity, causing long-term morbidity.

Neuroblastoma is a cancer of the sympathetic nervous system accounting for about 12% of cancer-related deaths in children under 15-years old. It is a heterogeneous disease in which up to 50% of patients have a high-risk behavior characterized by widespread dissemination and poor long-term survival, even when using intensive multimodal treatments. Significant improved outcomes were published nearly a decade ago with the use of myeloablative therapy with stem-cell rescue, followed by differentiation treatment with isotretinoin (Matthay, 1999).

However, over 50% of patients receiving standard therapy relapse and ultimately die from the tumor. Consequently, the major obstacle to cure, once remission is achieved, is the chemotherapy-refractory disease that eludes the current methods for its detection. This failure has led to a resurgence of interest in alternative methods of disease eradication. Immunotherapy became a particular and hopeful option.

## Discussion

### Experimental treatment: immunotherapy

**Figure 1:**
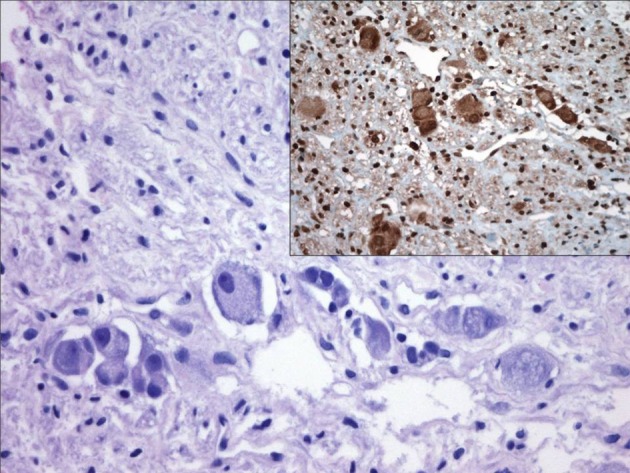
**Nests of maturing ganglion cells.** Inset: positivity of these cells for 14F7 ganglioside.

Our patient was eligible for our phase I study of the monoclonal anti-idiotype antibody racotumomab (formerly called 1E10), that targets NeuGc-containing gangliosides. In this case, biopsy specimens from the bone marrow showed marked positivity to the ganglioside antigen NeuGcGM3 (Figure 1) (Scursoni et al., 2011). As scheduled by the protocol, she received 3 intradermal applications of alum-adsorbed racotumomab at a dose of 0.15 mg each in the anterior left forearm. The drug was administered on an ambulatory basis every 14 days and the child presented only mild side effects such as localized painless erythema 3 cm in diameter at the injection site that appeared 8 h after application and disappeared within 24 h, without any treatment. No laboratory alterations or other evidence of toxicity was observed.

One month after the last monoclonal antibody dose, she had progressive disease in the orbital metastasis and complained of generalized bone pain and lower limb paresis. A nuclear magnetic resonance imaging (MRI) was performed revealing spinal cord compression. Because of that, local orbital and spinal radiotherapy was carried out for palliation. The patient died one month after this episode due to disease progression.

**Figure 2:**
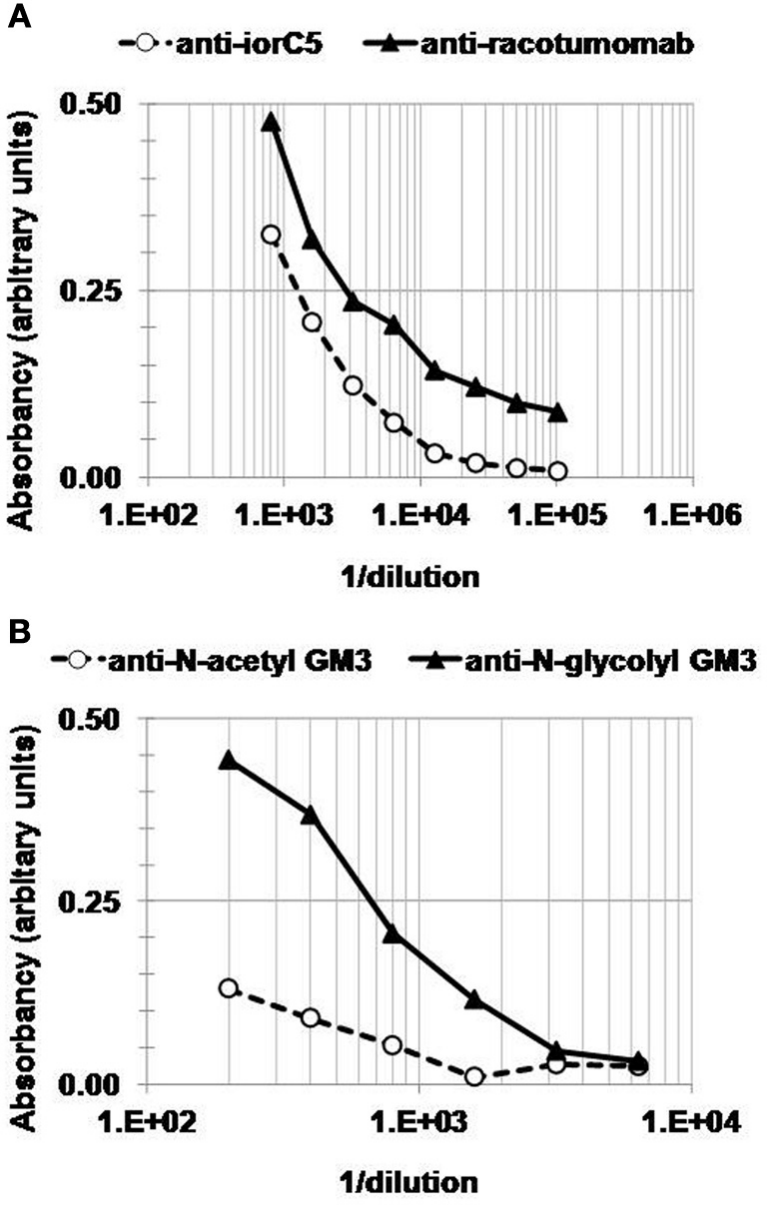
**Antibody response elicited by racotumomab immunization.** Serum samples obtained two weeks after the second immunization were assessed for anti-mouse **(A)** and anti-ganglioside **(B)** antibodies. **(A)** Plates were coated with either racotumomab or an isotype-matched monoclonal antibody (iorC5). Overlaid titration curves show a significantly stronger reactivity toward racotumomab. **(B)** Plates were coated with N-glycolyl GM3 or N-acetyl GM3. No significant binding to N-acetyl GM3 was observed.

Along with follow-up laboratory results, serum samples were drawn to assess the induction of antigen-specific antibodies. The patient developed a positive anti-racotumomab IgG response (Figure 2A). The reactivity against iorC5, an isotype-matched murine monoclonal antibody was significantly lower than that to racotumomab, underscoring the immunodominance of racotumomab idiotype. Most interestingly, anti-NeuGcGM3 IgM antibodies were also induced. The anti-ganglioside response was observed two weeks after the second racotumomab immunization (Figure 2B), but faded away later and was not detectable one month after the third immunization. The anti-ganglioside response was NeuGcGM3 specific, as no significant reactivity was observed against N-acetyl (NeuAc) GM3 (Figure 2B).

### Immunotherapy in neuroblastoma

Current conventional therapies against neuroblastoma have proven inadequate to treat advanced and refractory disease. The future success of immunotherapy against neuroblastoma should include the combination of treatment modalities for targeting minimal residual disease. Neuroblastoma is the archetypical pediatric tumor for the use of immunotherapy (Modak and Cheung, 2007). The most widely used immunotherapy for neuroblastoma consists of murine or humanized-murine chimeric monoclonal antibodies directed against GD2, a disialoganglioside expressed in tumors of neuroectodermal origin. However, GD2 is poorly immunogenic, so this antibody had to be combined with immunomodulators to elicit a significant response, which was also associated to severe toxicity (Gray and Kohler, 2009). This treatment is not available outside clinical trials, so it is not an option for patients with neuroblastoma in developing countries. There is good evidence that many children with neuroblastoma elicit an immune response against their tumor. Although these spontaneous immune responses are generally weak and fail to control tumor growth, they have the potential to be manipulated to provide a highly specific therapy for minimally disseminated neuroblastoma. Many different immunotherapy approaches have emerged over the last decades, but only a small number of these have achieved the level of clinical studies with relevant but arbitrary clinical responses, providing evidence that the immune system is capable, under certain conditions, of controlling and eradicating the tumor (Gray and Kohler, 2009).

GD2 is uniformly expressed in neuroblastomas (Wayne et al., 2010). Its function is not fully established but is thought to play a major role in tumor cell attachment to extracellular matrix proteins. GD2 expression in normal tissues of adults and children is restricted to the central nervous system, peripheral nerves, and skin melanocytes (Modak and Cheung, 2007). Due to the relatively tumor-selective expression combined with its presence on the cell surface, GD2 is an attractive target for this kind of therapy and immunotherapy with the humanized monoclonal antibody 14.18 showed improved results combined with high dose therapy, autologous stem cell rescue, and differentiation therapy in a randomized trial (Modak and Cheung, 2007).

Active specific immunotherapy is a promising field in cancer research. NeuGc gangliosides, particularly the NeuGcGM3 ganglioside, have received considerable attention as a privileged target for cancer therapy (Fernandez et al., 2010). They are usually undetectable in healthy human tissues and fluids, but widely expressed in tumor tissues, including neuroblastoma and other pediatric solid tumors (Fernandez et al., 2010). Glycosidic chains of gangliosides contain at least one sialic acid residue. Sialic acid may have some variations, with the most common versions in mammals: Neu-Ac and Neu-Gc. Many of these glycolipid compounds are abundantly expressed in tumor cells, being regarded as promising targets for immunotherapy, as is the case of the NeuGcGM3 monosialoganglioside.

Clinical trials have been carried out with murine monoclonal anti-idiotype racotumomab to treat adult tumors such as melanoma, breast, and lung cancer, which express surface NeuGc gangliosides. These studies showed a correlation between development of antibodies against NeuGcGM3 and increased survival time (Guthmann et al., 2006; Oliva et al., 2006; Hernandez et al., 2008; Fernandez et al., 2010). Our group reported the expression of these gangliosides in 85% of the cases of neuroblastoma studied (Scursoni et al., 2011). Thus, a Phase I clinical trial is currently underway at our hospital in pediatric patients with refractory or resistant neuroblastoma and other neuroectodermic tumors using racotumomab. This vaccine has not been previously used in children, so we hereby present a case report of the first patient recruited in the study who received this vaccine.

This Phase I clinical trial is being carried out in Garrahan Hospital in pediatric patients diagnosed with cancer expressing NeuGc gangliosides and who have been previously refractory or resistant to conventional cancer treatments. The main objective is to evaluate the acute toxicity and maximal tolerated dose and, secondly, immunological and clinical response to treatment with racotumomab. This is the first time that this monoclonal antibody is used in children. Since this is a treatment directed to minimally disseminated disease, it is probable that very few patients with refractory disease would present a clinical response. Racotumomab was immunogenic and induced a strong anti-mouse IgG response with marked specificity to racotumomab idiotype. Furthermore, a transient IgM response specific for NeuGcGM3 was also observed after the second administration of racotumomab. No anti-ganglioside IgG response was detected, which is in line with the need for an extended booster regimen to elicit the antibody class switch (Guthmann et al., 2006). The immunodominance of racotumomab idiotype and the NeuGcGM3 specificity of the anti-ganglioside response have been described earlier for adult patients under a 1 mg racotumomab immunotherapy regimen (Guthmann et al., 2006). The present results, obtained from a 4-year-old infant receiving a 0.15 mg dose-level, suggest that a similar immunogenicity might be elicited in infants, thereby warranting further investigation of racotumomab in pediatric cancer immunotherapy.

## Concluding remarks

Interest in immunotherapy for pediatric cancer is increasing. However, most therapies are still experimental. The challenges for the next decade involve translating pre-clinical treatment outcomes into effective treatments for patients and making these treatments available in developing countries where toxicity is a severe problem.

Racotumomab vaccination proved to elicit an immune response with a favorable toxicity profile. A Phase I trial is accruing patients to fully characterize its toxicity profile and it may become an innovative alternative for treatment of neuroblastoma and other embryonal pediatric malignancies.

### Conflict of interest statement

M. D. Guthmann is a Clinical Research Associate at Elea Laboratory, sponsor of phase I clinical trial described in this report. The remaining authors declare that they have no commercial or financial relationships that could be construed as a potential conflict of interest.
